# Comparison of Pandemic (H1N1) 2009 and Seasonal Influenza, Western Australia, 2009

**DOI:** 10.3201/eid1609.100076

**Published:** 2010-09

**Authors:** Dale Carcione, Carolien Giele, Gary K. Dowse, Donna B. Mak, Leigh Goggin, Kelly Kwan, Simon Williams, David Smith, Paul Effler

**Affiliations:** Author affiliations: Communicable Disease Control Directorate, Perth, Western Australia, Australia (D. Carcione, C. Giele, G.K. Dowse, D.B. Mak, L. Goggin, K. Kwan, P. Effler);; PathWest Laboratory Medicine, Nedlands, Western Australia, Australia (S. Williams, D. Smith)

**Keywords:** Viruses, respiratory infections, influenza, seasonal influenza, H1N1, pandemic, Australia, research

## Abstract

We compared confirmed pandemic (H1N1) 2009 influenza and seasonal influenza diagnosed in Western Australia during the 2009 influenza season. From 3,178 eligible reports, 984 pandemic and 356 seasonal influenza patients were selected; 871 (88.5%) and 288 (80.9%) were interviewed, respectively. Patients in both groups reported a median of 6 of 11 symptoms; the difference between groups in the proportion reporting any given symptom was <10%. Fewer than half the patients in both groups had >1 underlying condition, and only diabetes was associated with pandemic (H1N1) 2009 influenza (odds ratio [OR] 1.9, 95% confidence interval [CI] 1.1–3.5). A total of 129 (14.8%) persons with pandemic (H1N1) 2009 and 36 (12.5%) persons with seasonal influenza were hospitalized (p = 0.22). After controlling for age, we found that patient hospitalization was associated with pandemic (H1N1) 2009 influenza (OR 1.5; 95% CI 1.1–2.1). Contemporaneous pandemic and seasonal influenza infections were substantially similar in terms of patients’ symptoms, risk factors, and proportion hospitalized.

## Abstract 2

TOC summary: Infections were similar in terms of symptoms, risk factors, and proportion of patients hospitalized.

Pandemic (H1N1) 2009 influenza A emerged in Mexico in March 2009 and was first reported in the United States the following month, toward the close of the 2008–09 influenza season in the Northern Hemisphere (*1**,**2*). The virus rapidly spread worldwide, with the first pandemic (H1N1) 2009 infection reported in Australia on May 9, 2009, just before the start of the traditional winter influenza season in the Southern Hemisphere (*3*).

There are little data directly comparing confirmed pandemic (H1N1) 2009 with contemporaneous seasonal influenza over the same influenza season (*4**–**6*). Many of the reports on the epidemiology of influenza in 2009 to date have focused exclusively on pandemic (H1N1) 2009 or have used limited laboratory-based surveillance data on isolation rates for seasonal and pandemic (H1N1) 2009 influenza viruses (*7**–**13*). Other reports have compared pandemic (H1N1) 2009 and seasonal influenza infections that occurred outside the usual influenza season (*14*). Still other investigators have compared various indicators of influenza severity during the current pandemic with historical data from previous annual influenza epidemics (*15**,**16*). Interpretation of such comparisons is challenging because of variation in influenza activity from season to season. Furthermore, heightened awareness surrounding the current pandemic may have affected patient care-seeking behavior or physician diagnostic practices, thus potentially creating bias in year-to-year comparisons. Examining confirmed pandemic (H1N1) 2009 and seasonal influenza infections occurring in the same population during the 2009 influenza season enables a more straightforward comparison.

We interviewed persons with laboratory-confirmed pandemic (H1N1) 2009 or seasonal influenza infection over a 10-week period encompassing the peak of the winter influenza season. This effort enabled us to directly compare the clinical illness and predisposing medical risk factors associated with pandemic (H1N1) 2009 and seasonal influenza infections diagnosed contemporaneously from the general population of Western Australia, which has a population of 2.2 million persons.

## Methods

All clinical laboratories report positive influenza test results to the Communicable Disease Control Directorate (CDCD), Department of Health, Western Australia. For diagnosis, respiratory samples, usually combined nose and throat swab specimens, were tested by PCR. More than 90% of the specimens were tested at PathWest Laboratory Medicine Western Australia, Queen Elizabeth II Medical Centre, by using an assay that identified and distinguished between pandemic (H1N1) 2009 and seasonal influenza A/H1, A/H3, and B (*17*); positive results for pandemic (H1N1) 2009 influenza virus reported from other clinical laboratories were considered as single infections with pandemic (H1N1) 2009.

Reports of all PCR-confirmed influenza infections were reviewed. Patients were excluded if the results could not differentiate between pandemic and seasonal viruses or if the patient was identified as infected with pandemic (H1N1) 2009 and seasonal influenza. Pandemic influenza was defined as PCR-confirmed pandemic (H1N1) 2009 influenza infection, and seasonal influenza was defined as any PCR-confirmed influenza infection for which infection with pandemic (H1N1) 2009 virus had been excluded.

**Figure 1:**
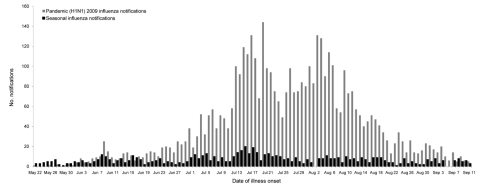
Number of notifications for pandemic and seasonal influenza, by date of onset and type, Western Australia, May 22–September 11, 2009. Influenza subtypes reported during the study period (n = 3,178): pandemic (H1N1) 2009, 2,794 (87.9%); influenza A (H3N2), 253 (8.0%); seasonal influenza A (H1N1), 89 (2.8%); influenza B, 36 (1.1%); and seasonal influenza A (not subtyped), 6 (0.2%).

The study began May 29, 2009 (1 week after the illness onset in the first person with confirmed pandemic [H1N1] 2009 influenza infection in Western Australia) and concluded August 7, 2009 (Figure 1) (*18*). From the study inception through July 13, 2009, attempts were made to interview every patient with confirmed influenza illness reported to CDCD. On July 14, 2009, we instituted a sampling framework because of increasing numbers of reported infections. The sampling scheme entailed identifying the last digit of a sequentially-assigned identification number from the first patient reported each day with seasonal or pandemic (H1N1) 2009 influenza, then selecting all patients reported that day with the same last digit. If <20 patients were identified for interview, we added 1 to the digit (n + 1) and selected additional patients by using the same procedure. This process was repeated until up to 20 persons with seasonal influenza and 20 with pandemic influenza were chosen each day. If <20 influenza infections were reported on a given day for either seasonal or pandemic (H1N1) 2009 influenza, we attempted to interview all patients reported on that day.

Study participants were interviewed by a trained nurse who used a standard questionnaire. If a patient was unable to answer questions or was <18 years of age, the nurse interviewed a parent or other family member familiar with the patient’s situation. We made 6 attempts to contact the patient or a proxy, after which the patient was considered not contactable. Diagnostic specimens of participating patients were collected a median of 2 d after illness onset (interquartile range [IQR] 1–3 d), and patients were interviewed a median of 6 d after onset (IQR 5–8 d).

The patient’s self-reported symptoms, treatment with antiviral medications, presence of underlying medical conditions, and disease disposition were recorded. Information on hospitalization was obtained at the time of the interview and by retrospectively querying a hospital discharge database that covers all public hospitals in the state and 1 major private metropolitan facility. A cross-check with the hospital discharge database was performed for every influenza notification received at CDCD.

For our analysis, we first characterized all patients with pandemic (H1N1) 2009 or seasonal influenza infection reported to CDCD during the study period (the target population) in terms of age, sex, and hospitalization status by using univariate Mantel-Haenszel χ^2^ tests for proportions and *t* tests for population means. We then compared patients who were interviewed (the study population) with the remaining patients not interviewed in the target population in terms of age, sex, and hospitalization status; if a significant difference was identified, we weighted the interview responses from the study participants to reflect the target population (*19*). Finally, by using the data obtained during interviews, we performed univariate analyses to compare patients with pandemic influenza with those with seasonal influenza with respect to reported symptoms, underlying medical conditions, and treatment. Because the age structure of the population with pandemic influenza differed from that with seasonal influenza, we also computed odds ratios (ORs) for individual symptoms or underlying medical conditions by using logistic regression to control for age. In each of the regression analyses, the dependent variable was defined as influenza type (pandemic/seasonal), and the independent variables were limited to age in years and 1 dichotomous variable representing the presence or absence of a single patient characteristic (e.g., a symptom or underlying medical condition).

To assess whether antiviral medications might have influenced the symptoms reported, we performed a subanalysis restricted to patients who were treated with antiviral agents within the first 2 days of illness onset (early use of antiviral drugs) and compared those patients with patients who were never treated, controlling for age, influenza type, and the presence of underlying medical conditions.

Influenza-like illness was defined as documented fever >38°C or a history of fever when the temperature was not known, and cough or sore throat, or both. Risk difference was defined as the absolute difference in the proportion of pandemic and seasonal influenza patients reporting a given parameter. A p value <0.05 was considered significant. Statistical analyses were performed by using Epi Info 2000 (Centers for Disease Control and Prevention, Atlanta, GA, USA).

## Results

### Characteristics of the Target Population

**Figure 2:**
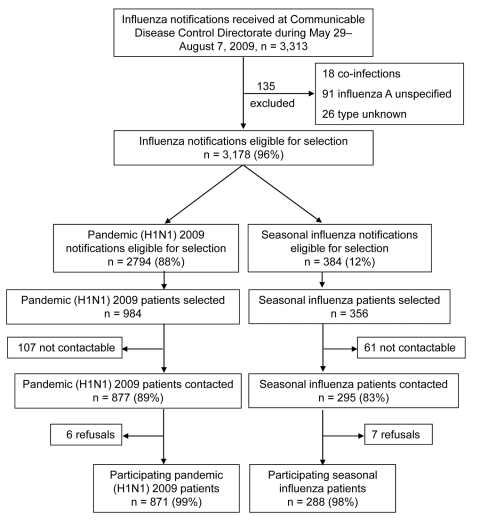
Recruitment of pandemic and seasonal influenza study participants, Western Australia, 2009.

A total of 3,313 notifications of laboratory-confirmed influenza were received at CDCD during the study period. Of these notifications, 117 (3.5%) were excluded because information on the viral strain and/or subtype was incomplete, and 18 (0.5%) were excluded because pandemic and seasonal influenza viruses were detected (Figure 2). Of the remaining 3,178 influenza infections reported, 2,794 (87.9%) were pandemic (H1N1) 2009 influenza and 384 (12.1%) were seasonal influenza. The proportion of each influenza subtype identified is shown in the inset in Figure 1. The mean age of patients with pandemic influenza was significantly lower than that for patients with seasonal influenza, 27 and 35 years, respectively (p<0.005).

Most of the pandemic and seasonal influenza patients were female, but the proportion of female patients among the seasonal influenza patients was significantly greater than that among patients with pandemic influenza, 57.3% (220/384) and 51.2% (1,431/2,794), respectively (p = 0.03 by χ^2^ test). However, the association between female sex and seasonal influenza was not significant when we controlled for age (p = 0.09).

A total of 415 (14.9%) of the 2,794 patients with pandemic influenza and 48 (12.5%) of the 384 patients with seasonal influenza were hospitalized. The difference between the proportion of patients hospitalized with pandemic and seasonal influenza was not significant on univariate analysis (p = 0.22, by χ^2^ test). However, when we controlled for age, the odds of hospitalization were significantly greater for persons with pandemic influenza (OR 1.53, 95% confidence interval [CI] 1.10–2.13; p = 0.011).

### Selection and Representativeness of Study Participants

A total of 984 patients with pandemic (H1N1) 2009 influenza and 356 patients with seasonal influenza were selected for interview, and 871 (88.5%) and 288 (80.9%) of selected patients completed the interview, respectively (Figure 2). Of the 181 patients selected but not interviewed, 168 were not able to be contacted because they did not have a working telephone number or did not answer after 6 attempts, and 13 declined to participate.

Patients who completed interviews were very similar to the remaining notified influenza patients who were not interviewed with respect to age and sex. The median age was 25 years (IQR 13–42 years) for study participants and 25 years (IQR 14–39 years) for the remaining notified influenza patients who were not interviewed. Women and girls accounted for 51.0% (591/1,159) of the study participants and 52.5% (1,060/2,019) of the remaining patients with notified influenza cases who were not interviewed (p = 0.41, by χ^2^ test).

Hospitalized persons were underrepresented among the study participants compared with the remaining influenza case-patients who were not interviewed, i.e., 11.9% (138/1,159) of the interviewed patients had been hospitalized compared with 16.1% (325/2,019) of the patients not interviewed (p<0.05). The interview data were therefore weighted to reflect the hospitalization rate in the target population for both pandemic and seasonal influenza.

### Comparison of Pandemic and Seasonal Influenza in the Study Population

**Figure 3:**
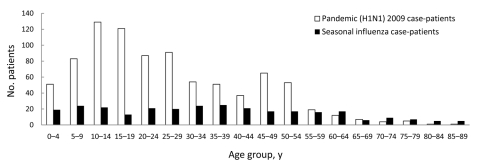
Age distribution for study participants, by influenza type, Western Australia, 2009.

The age distribution for study participants, by influenza type, is shown Figure 3. As in the target population, the mean age of study participants with pandemic (H1N1) 2009 influenza was significantly younger than the mean age of study participants with seasonal influenza, 26 and 36 years, respectively (p<0.005). Only 6% (49/871) of the study participants with pandemic influenza were >55 years of age compared with 23% (65/288) of those with seasonal influenza (p<0.005).

**Figure 4:**
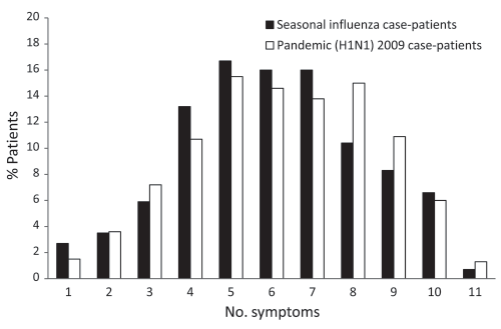
Number of symptoms reported by study participants with influenza, by influenza type, Western Australia, 2009.

The distribution of the total number of symptoms reported by each patient with pandemic or seasonal influenza is shown in Figure 4. Patients with pandemic influenza and seasonal influenza reported a median of 6 symptoms (IQR 5–8 symptoms and 4–8 symptoms, respectively). When we controlled for age, no significant association was found between influenza type and the total number of symptoms that patients reported (p = 0.19).

The number and proportion of patients reporting specific symptoms are presented in the Table. The difference in the proportion reporting a given symptom between patients with pandemic and seasonal influenza was <10% for all symptoms. Univariate analyses showed that fever and diarrhea were significantly more common for patients with pandemic influenza. Controlling for age added cough and myalgia/arthralgia to the symptoms significantly associated with pandemic (H1N1) 2009 influenza compared to those with seasonal influenza. Rhinorrhea was significantly associated with seasonal influenza on univariate analysis, and this association persisted when controlling for age.

**Figure 5:**
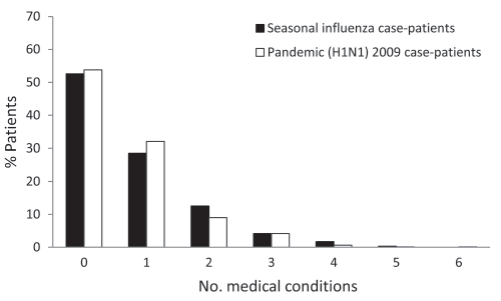
Number of underlying medical conditions reported by study participants, by influenza type, Western Australia, 2009.

The distribution of the total number of underlying medical conditions reported by individual patients with pandemic (H1N1) 2009 or seasonal influenza is shown in Figure 5. Just over half of the patients with seasonal or pandemic influenza had no underlying medical condition(s).

The proportion of patients who reported a specific underlying medical condition is presented in the Table. The absolute difference in the proportion of patients that reported a given medical condition between those with pandemic (H1N1) 2009 and seasonal influenza was greatest for pregnant patients but still <5% for all underlying conditions queried. None of the 11 underlying medical conditions we queried were significantly associated with pandemic or seasonal influenza in univariate analysis. When controlling for age, we found that only the odds of reporting a history of diabetes were significantly greater among patients with pandemic influenza (OR 1.93, 95% CI 1.07–3.51; p = 0.03).

Having >1 underlying medical condition was not significantly associated with pandemic influenza in the univariate analyses. However, after we controlled for age, we observed that the odds of reporting >1 underlying medical conditions were significantly greater among patients with pandemic influenza (Table).

**Table:** Symptoms, underlying medical conditions, and medical care reported by study participants in a comparison of pandemic (H1N1) 2009 and seasonal influenza, Western Australia, 2009*

Parameter	No. respondents	Pandemic (H1N1) 2009, no. (%)	Seasonal influenza, no. (%)	RD	Univariate χ^2^ p value	OR† (95% CI)	p value
Symptoms							
Fever‡	1,159	762 (88)	225 (78)	10	0.001	1.64 (1.15–2.35)	0.01§
Cough	1,159	743 (85)	236 (82)	3	NS	1.45 (1.01–2.34)	0.01§
Myalgia/arthalgia	1,159	565 (65)	173 (60)	5	NS	1.40 (1.06–1.87)	0.02§
Diarrhea	1,159	165 (19)	35 (12)	7	0.008	1.72 (1.15–2.57)	0.01§
Rhinorrhea	1,159	494 (57)	189 (66)	−9	0.007	0.60 (0.45–0.80)	0.01§
Sore throat	1,159	488 (56)	169 (59)	**−**3	NS	0.82 (0.62–1.09)	0.17
Shortness of breath	1,159	289 (33)	99 (35)	**−**2	NS	1.14 (0.85–1.53)	0.38
Headache	1,159	537 (62)	176 (61)	1	NS	1.02 (0.77–1.35)	0.91
Vomiting or nausea	1,159	284 (33)	80 (28)	5	NS	1.14 (0.84–1.54)	0.40
Fatigue	1,159	639 (73)	205 (71)	2	NS	1.12(0.83–1.51)	0.47
Rigors	1,159	471 (54)	148 (52)	2	NS	1.13 (0.86–1.48)	0.40
ILI criteria met¶	1,159	706 (81	209 (73)	8	0.002	1.50 (1.09–2.06)	0.01§
Underlying medical conditions							
Diabetes	1,032	49 (7)	18 (6)	1	NS	1.93 (1.07–3.51)	0.03§
Heart disease	1,027	34 (5)	20 (7)	**−**2	NS	1.16 (0.63–2.16)	0.63
Respiratory disease	1,031	178 (24)	62 (22)	2	NS	1.33 (0.94–1.87)	0.10
Renal disease	1,028	13 (2)	7 (2)	0	NS	1.17 (0.44–3.10)	0.76
Neurologic disease	1,028	12 (2)	7 (2)	0	NS	0.91 (0.33–2.53)	0.86
Hematologic disorder	1,028	19 (3)	5 (2)	1	NS	2.33 (0.82–6.66)	0.11
Metabolic disease (not diabetes)	1,028	12 (2)	4 (1)	1	NS	1.25 (0.38–4.06)	0.71
Immune impairment	1,028	26 (3)	16 (6)	**−**3	NS	0.88 (0.45–1.71)	0.70
Morbid obesity	1,031	64 (9)	32 (11)	**−**2	NS	1.12 (0.70–1.80)	0.64
Current smoker	1,032	98 (13)	35 (12)	1	NS	1.36 (0.89–2.08)	0.16
Pregnancy (women only)	556	36 (9)	8 (5)	4	NS	1.85 (0.84–4.10)	0.13
Any	1,051	366 (48)	135 (47)	1	NS	1.44 (1.07–1.94)	0.02§
Medical care							
Hospitalization	1,159	129 (15)	36 (12)	3	NS	1.58 (1.04–2.39)	0.03§
Antiviral treatment	1,103	388 (47)	71 (26)	21	0.001	3.12 (2.27–4.29)	0.01§

*Totals respondents may not sum to 1,159 for all parameters because questions regarding underlying medical conditions and antiviral treatment were added shortly after the study was initiated, and there are intermittent missing values to individual questions for some respondents. RD, risk difference (absolute difference in the proportion of pandemic and seasonal influenza patients reporting a given parameter); OR, odds ratio; CI, confidence interval; NS, not significant; ILI, influenza–like illness (patient had fever and cough or sore throat). †ORs were computed by using logistic regression to control for age. Each row depicts data from a separate regression equation, where the dependent variable was defined as influenza type and age (in years) and a single patient characteristic, as listed in the first column of the row (coded as a dichotomous variable indicating the presence or absence of the respective symptom or underlying medical condition) were included as the predictor variables. In all of the logistic analyses performed, age remained significantly associated with influenza type, i.e., younger patients had a higher odds of having pandemic influenza compared with seasonal influenza. ‡Fever was defined as temperature >38°C or subjective fever if temperature was not measured. §Significant OR obtained using logistic regression. ¶ Patient reported >1 of the underlying medical conditions listed.

**Figure 6:**
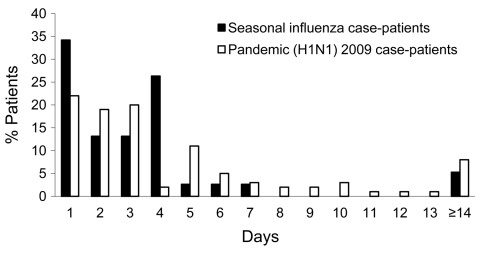
Duration of hospital stay for study participants, by influenza type, Western Australia, 2009.

By design, the analysis that used weighted data shown in the Table mirrors the hospitalization rates observed for pandemic and seasonal influenza in the target population (14.9% vs. 12.5%, p = 0.22). The distribution of the length of stay for hospitalized study participants is shown in Figure 6. The mean duration of hospitalization was 5.1 d (median 3 d, IQR 2–6 d) for patients with pandemic influenza and 3.4 d (median 2 d, IQR 1–4 d) for those with seasonal influenza (p = 0.13). Although the findings were not significant, a trend toward longer hospital stays did appear for those with pandemic (H1N1) 2009 versus seasonal illness, based on the proportion of patients hospitalized an additional >7 d (21% vs. 8%, p = 0.07).

Of the 10 patients hospitalized for >14 d, all had >1 underlying medical condition, and 6 had >2 conditions. Two patients in the study population died; both had pandemic (H1N1) 2009.

The proportion of patients reporting treatment with antiviral medication was significantly greater among those with pandemic influenza (Table). Information on the type of antiviral drug received was recorded for 427 (94.9%) of 450 patients treated with antiviral drugs; 426 reported taking oseltamivir and 1 reported taking zanamivir. The mean lag time between illness onset and starting antiviral treatment was 2.7 d (median 2 d, IQR 1–3 d) for patients with pandemic influenza and 2.3 d (median 2 d, IQR 1–3 d) for patients with seasonal influenza (p = 0.39).

Comparing patients given antiviral medications with those who were not, we found a significant inverse relationship (i.e., a protective effect) between early antiviral drug use and reported rhinorrhea for patients with pandemic influenza (OR 0.5, 95% CI 0.4–0.8; p = 0.005). We also observed a positive association between early antiviral drug use and nausea and vomiting; this association was robust and persisted when the analysis was simultaneously controlled for age, influenza type, underlying conditions, as well as other symptoms commonly associated with influenza and/or gastrointestinal illness (i.e., fever, cough, sore throat, diarrhea) (OR 1.6, 95% CI 1.2–2.1; p = 0.02). No other symptoms were associated, positively or negatively, with antiviral drug use in the first 2 days of illness onset.

## Discussion

This comparison of >1,000 total confirmed seasonal and pandemic (H1N1) 2009 influenza infections occurring contemporaneously over the peak of the traditional influenza season yielded several findings. First, the spectrum of clinical illness due to pandemic influenza was similar to that caused by seasonal influenza. Although several symptoms were more common in patients with pandemic influenza, the differences were modest and of limited clinical importance.

Our findings generally parallel those from a recent comparative analysis in Singapore, with some differences. For example, in Singapore, the proportions of patients with seasonal and pandemic influenza who reported diarrhea were 0% and 4%, respectively; these figures are substantially lower than those found in our study (12% and 19%, respectively), despite the fact that in both settings most nonpandemic influenza viruses identified were influenza A (H3N2). These differences highlight the need to consider data from diverse geographic, cultural, and healthcare environments when characterizing the clinical manifestations of influenza.

Second, we observed that the hospitalization rates for pandemic (H1N1) 2009 and seasonal influenza infections were similar. Our ability to use a comprehensive statewide database to identify hospital admissions in the broader target population permitted a robust analysis that found the overall proportion of confirmed pandemic and seasonal illnesses hospitalized was not significantly different when aggregated data were used in univariate analyses (p>0.05). However, if the analysis was controlled for age, the odds of being hospitalized were significantly greater for the population with pandemic influenza. These seemingly dissonant results actually reflect the fact that for many age groups there was a higher risk for hospitalization with pandemic (H1N1) 2009, but because patients with seasonal influenza were older relative to those with pandemic influenza and elderly patients are more likely to be admitted to hospital when ill with influenza, the cumulative hospitalization rate in the 2 patient groups was similar.

In addition, in this study, the mean duration of hospitalization was not statistically different between patients with pandemic (H1N1) 2009 and seasonal influenza even though other indicators suggested pandemic patients were hospitalized for longer periods. An analysis of a larger sample of hospitalized patients is underway.

Third, the underlying medical conditions associated with pandemic (H1N1) 2009 and seasonal influenza illnesses diagnosed in the community were nearly identical in terms of the type and number of conditions reported. Most patients in both groups reported no risk factors, and only when we controlled for age did we find an association between having >1 underlying medical condition and pandemic influenza. Notably, the largest risk difference we observed was for was pregnancy (4%). Univariate analyses showed that the association between pregnancy and pandemic influenza approached statistical significance (p = 0.08; analysis not shown). When we restricted our analysis to women 15–45 years of age, the risk difference nearly doubled, but significance was still not attained, perhaps as a consequence of the smaller sample size. Seasonal influenza is a well-established cause of serious illness during pregnancy, and several reports indicate that the risk for severe illness from pandemic (H1N1) 2009 may be even greater (*20**–**23*).

Obesity, newly recognized as a risk factor for severe influenza illness during the 2009 pandemic, was reported as often by patients with seasonal influenza as by those with pandemic influenza (11% vs. 9%; p>0.05). This finding suggests that obesity may be equally important as a risk factor for seasonal and pandemic (H1N1) 2009 (*24*).

Finally, because our study was not a randomized controlled trial, inferences about the effect of antiviral medications should be viewed with caution. For example, our observation that antiviral drug use was negatively associated with reported rhinorrhea may be due to the effect of treatment or may have resulted from a relative disinclination of providers to prescribe antiviral drugs for patients with rhinorrhea, on the basis of an assumption that nasal symptoms make influenza infection less likely (*25*). However, the robust positive association we observed in our population between antiviral agent use and nausea/vomiting suggests that there was a causal relationship, a conclusion consistent with that of a recent metaanalysis on oseltamivir use (*26*).

The limitations of our study include the following: reported underlying medical conditions were not objectively verified, data on the duration of symptoms were not collected, and interviewers were not blinded to influenza type when administering the questionnaire. Also, because this was a public health evaluation of notified influenza infections principally detected through routine healthcare practices in the community at large, we were unable to control for potential biases stemming from who was tested and who was not. However, because the healthcare provider could not be confident of whether the patient had pandemic (H1N1) 2009 or seasonal influenza at the time of testing, any bias in who was selected for testing should be approximately equal for pandemic (H1N1) 2009 and seasonal influenza patient groups. Lastly, a limitation inherent in the case–control study design we used was that we are unable to assess the extent to which the underlying medical conditions reported increased the risk for a diagnosis of influenza of either type, when compared with persons without underlying medical conditions.

In summary, our head-to-head comparison of confirmed pandemic (H1N1) 2009 and contemporaneous seasonal influenza infections found little to differentiate the 2 in terms of symptoms, underlying medical conditions, and the proportion of patients hospitalized. These results add to the growing body of knowledge about pandemic (H1N1) 2009 and are in general agreement with several studies that used different methods in other settings (*27*). These data are important because early in the pandemic some reports espoused different conclusions; 1 report estimated the lethality of pandemic (H1N1) 2009 to be ≈1 death per 10,000 infections, about 100× greater than that for regular seasonal influenza (*28**,**29*)*.* Worldwide, unprecedented levels of resources have been expended to mitigate the impact of pandemic (H1N1) 2009. In the United States alone, the federal government appropriated $7.65 billion for this effort (*30*). This commitment to controlling pandemic (H1N1) 2009 is to be lauded, but we must not lose sight of the fact that seasonal influenza remains an important, albeit relatively uncelebrated, cause of illness and death each year. As the pandemic (H1N1) 2009 response draws to a close, it may be prudent to revisit the level of effort directed toward reducing the enormous effects, in terms of costs and health outcomes, associated with annually recurring influenza epidemics (*31*).
